# Genome analysis suggests the bacterial family *Acetobacteraceae* is a source of undiscovered specialized metabolites

**DOI:** 10.1007/s10482-021-01676-7

**Published:** 2021-11-10

**Authors:** Juan Guzman, Andreas Vilcinskas

**Affiliations:** 1 Department of Bioresources, Fraunhofer Institute for Molecular Biology and Applied Ecology, Ohlebergsweg 12, 35392 Giessen, Germany; 2 Institute for Insect Biotechnology, Justus-Liebig-University of Giessen, Heinrich-Buff-Ring 26-32, 35392 Giessen, Germany

**Keywords:** *Acetobacteraceae*, Phylogeny, Biosynthesis, Specialized metabolites

## Abstract

*Acetobacteraceae* is an economically important family of bacteria that is used for industrial fermentation in the food/feed sector and for the preparation of sorbose and bacterial cellulose. It comprises two major groups: acetous species (acetic acid bacteria) associated with flowers, fruits and insects, and acidophilic species, a phylogenetically basal and physiologically heterogeneous group inhabiting acid or hot springs, sludge, sewage and freshwater environments. Despite the biotechnological importance of the family *Acetobacteraceae*, the literature does not provide any information about its ability to produce specialized metabolites. We therefore constructed a phylogenomic tree based on concatenated protein sequences from 141 type strains of the family and predicted the presence of small-molecule biosynthetic gene clusters (BGCs) using the antiSMASH tool. This dual approach allowed us to associate certain biosynthetic pathways with particular taxonomic groups. We found that acidophilic and acetous species contain on average ~ 6.3 and ~ 3.4 BGCs per genome, respectively. All the *Acetobacteraceae* strains encoded proteins involved in hopanoid biosynthesis, with many also featuring genes encoding type-1 and type-3 polyketide and non-ribosomal peptide synthases, and enzymes for aryl polyene, lactone and ribosomal peptide biosynthesis*.* Our in silico analysis indicated that the family *Acetobacteraceae* is a potential source of many undiscovered bacterial metabolites and deserves more detailed experimental exploration.

**Supplementary Information:**

The online version contains supplementary material available at 10.1007/s10482-021-01676-7.

## Introduction

*Acetobacteraceae* is an economically important family of bacteria, with several strains used for industrial biotechnology applications including the commercial production of vinegar and fermented foods, bacterial cellulose, and sorbose, a key precursor of vitamin C (Lynch et al. 2019; Murooka 2016; Pappenberger and Hohmann 2014). The family is divided into two groups: acetous and acidophilic species (Hördt et al. 2020; Komagata et al. 2014). Acetous species are also known as acetic acid bacteria (AAB) and most can transform ethanol into acetic acid, although there are some exceptions such as *Asaia* spp. (Malimas et al. 2017). AAB are typically found in flowers, fruits and other sugary organs of plants, and in traditional vinegars and other fermentation products (Yamada 2016), although some have recently been shown to consistently associate with insects (Guzman et al. 2021; Li et al. 2015; Roh et al. 2008). Acidophilic species appear to be phylogenetically more distant from the AAB (Hördt et al. 2020), and show diverse phenotypes and adaptations, including acidophilic, neutrophilic, thermophilic and phototrophic characteristics (Komagata et al. 2014). This group has been isolated from paddy soils, acid or hot springs, soil crust, sludge, sewage, freshwater ponds, air-conditioning systems, and certain *Roseomonas* strains have even been isolated from human patients (Dé et al. 2004; Sievers and Swings 2015). The *Acetobacteraceae* currently includes 44 genera and 177 valid species, split into 19 genera and 97 species of AAB, and 25 genera and 80 species of acidophilic bacteria (Parte et al. 2020). The family belongs to the order *Rhodospirillales*, class *Alphaproteobacteria*, and their closer siblings are the recently proposed families *Stellaceae* and *Reyranellaceae*, based on phylogenomic and phenotypic analysis (Hördt et al. 2020). Some species currently classified as acidophilic bacteria are likely to be assigned to new families in the future when more genomic data become available. No specialized metabolites (< 2 kDa) have been reported thus far from any member of the family *Acetobacteraceae*, but it remains an untapped potential source of natural products given that related taxa appear to carry tens of biosynthetic gene clusters (BGCs) based on wide genomic analysis (Mukherjee et al. 2017).

The production of specialized metabolites has been intensively studied in streptomycetes and myxobacteria because they are known producers of antibiotics. The production of metabolites depends on the ecological context, in which the synthesized compounds confer competitive advantages to the producer, overcoming the energy costs of maintaining the BGCs (Hoskisson and Fernández-Martínez 2018; Jensen 2016). BGCs often encode not only enzymes but also other essential complementary proteins such as assembly scaffolds, metabolite resistance factors and regulatory effectors. Computational methods have been developed to identify the presence of BGCs in the exponentially growing resource of microbial genomic data (Medema et al. 2021; Medema and Fischbach 2015). The standard tool for this purpose is antiSMASH, which interrogates the protein sequences encoded in the genomes for sequence similarity to a library of hidden Markov models extracted from core biosynthetic proteins (Medema et al. 2011). The cluster boundaries are expanded to include other nearby core proteins, and accessory proteins in the vicinity are detected (Blin et al. 2017b). The search is finalized by evaluating the similarity of the detected gene set to known BGCs. One of the main limitations of library-based genome mining is that it detects proteins similar to known biosynthetic proteins but excludes unknown proteins that might produce entirely new molecules (Blin et al. 2017a). Here we took 127 published genomes of *Acetobacteraceae* type strains and used them for phylogenetic analysis and genome mining in order to find correlations between cladistics and the conservation of certain specialized biosynthetic traits. Our results will help to focus discovery efforts on bacterial producers of novel metabolites with potential applications in the pharmaceutical and agrochemical industries.

## Materials and methods

### Genomic dataset

The genome sequences of 141 bacterial type strains (Supplementary Table 1) were downloaded from NCBI GenBank in September 2021. Only genomic assemblies with sufficient quality (N50 > 50 kb) were included in the study. The dataset comprised all 139 genomes available for *Acetobacteraceae* type strains and two type species as outgroups, namely *Azospirillum lipoferum* 59b^T^ and *Skermanella aerolata* KACC 11604^T^. The dataset included 43 acidophilic strains and 96 acetous strains.

### Phylogenetic analysis

The protein sequences were annotated from the downloaded genomes using Prokka v1.14.5 (Seemann 2014). Homologous protein sequences between species were identified using hmmsearch v3.2 (Eddy 2011) with hidden Markov models defined in bcgTree v1.1.10 (Ankenbrand and Keller 2016). The hits were aligned using Muscle v3.8.31 (Edgar 2004) and the unaligned sequences were trimmed using Gblocks v0.91b (Talavera and Castresana 2007). A concatenated alignment of 50 homologous proteins present as single copies in all species was generated, comprising the sequences of DnaN, DnlJ, Era, Frr, GrpE, InfC, LepA, NusA, PrfA, PyrG, RbfA, RecA, SecY, l-Gly-tRNA and l-Thr-tRNA ligases, and ribosomal proteins L1, L2, L3, L4, L5, L6, L9, L10, L12, L13, L14, L16, L17, L19, L20, L23, L24, L27, L29, L28, S2, S3, S4, S7, S6, S8, S9, S11, S12, S15, S16, S17, S18, S19 and S20. The tree was inferred using ExaBayes v1.5.1 (Aberer et al. 2014) as a consensus model built from four independent runs estimated over 10^6^ generations. A tree with exactly the same topology and similar branch support values was generated from the alignment using IQ-Tree v2.1.3 (Minh et al. 2020). The best model search for each partitioned protein sequence was performed using ModelFinder (Kalyaanamoorthy et al. 2017) and bootstrap values were calculated over 10^5^ replicates using ultrafast bootstrap approximation (Hoang et al. 2017).

### Biosynthetic gene clusters

BGCs were predicted on the same dataset of 139 *Acetobacteraceae* type strains. The downloaded genomes were analyzed using the antibiotics and secondary metabolite analysis shell (antiSMASH) v5.2.0 (Blin et al. 2019) website in relaxed mode with all the extra features selected. Each identified region was counted and classified according to the biosynthetic core and accessory genes. BLAST analysis of the protein sequences against the GenBank non-redundant protein sequences database (340.8 million entries) was used to infer functional annotations for certain core and accessory proteins. The files in gbk format were downloaded from antiSMASH and used to assess gene cluster synteny and protein–protein similarity using Clinker v0.0.12 (Gilchrist and Chooi 2020) based on the BGCs for known compounds in the MIBIG repository (Kautsar et al. 2019).

## Results and discussion

### GC content and genome size

**Fig. 1:**
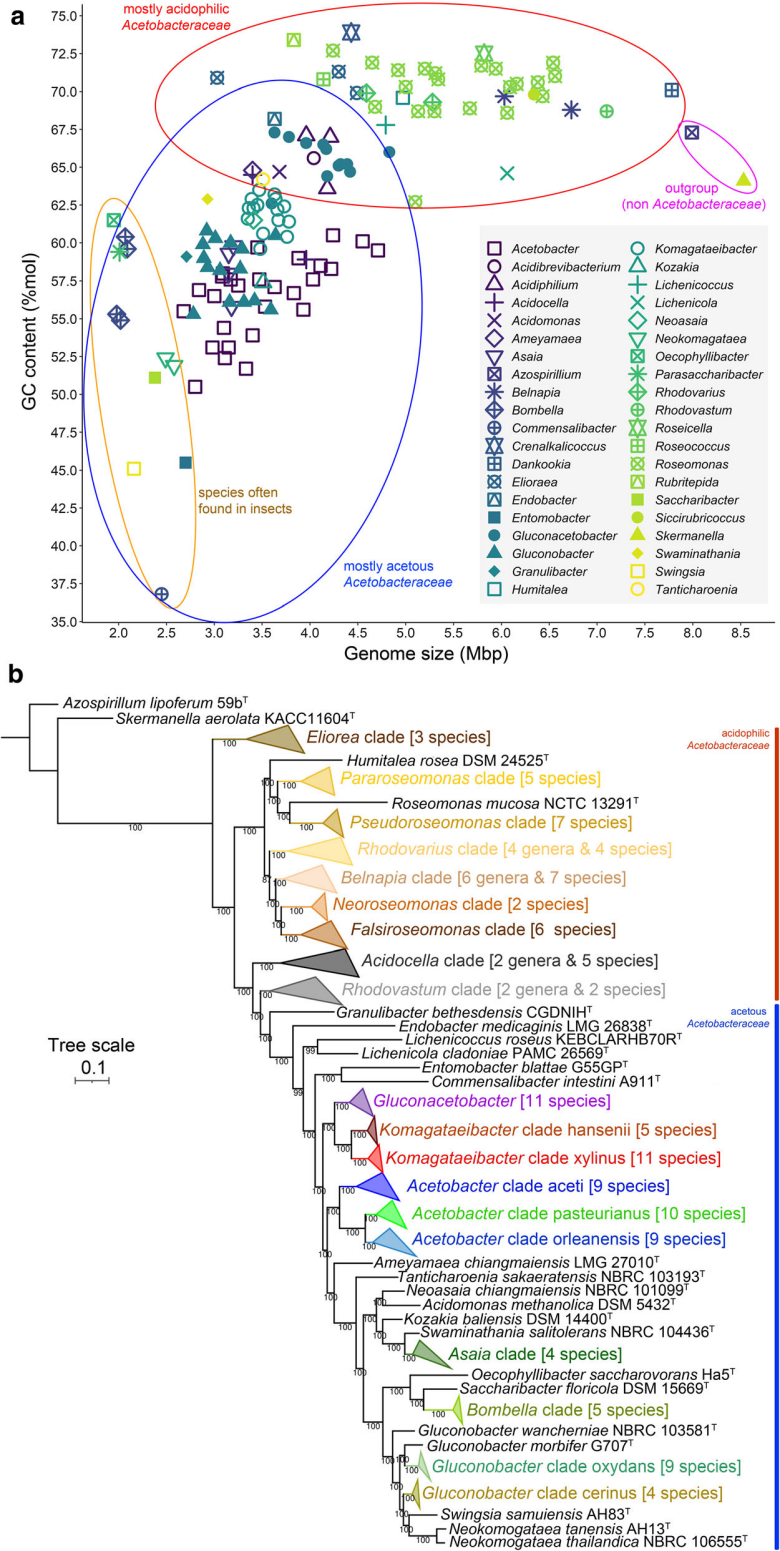
GC content *vs* genome size plot and phylogenomic tree for *Acetobacteraceae* type strains **a** GC content and genome size plot grouping the type strains from each genus under the same symbol. The strains *Azospirillum lipoferum* 59b^T^ and *Skermanella aerolata* KACC 11604^T^ are used as outgroups for the family *Acetobacteraceae*. The plot reveals three groups of bacteria with some degree of overlap, globally differentiated as mostly acidophilic, mostly acetous, and acetous species often associated with insects. **b** Phylogenomic tree inferred from 50 housekeeping protein sequences showing the two different groups of the family *Acetobacteraceae*. The topology of the tree is supported by both Bayesian and maximum likelihood inference methods. Distinct clades (based on monophyly and a shorter branch length distance) were proposed particularly for the acetous group. The species organization into clades is detailed in Supplementary Table 2

A graphic plot examination of the variation in GC-content and genome size values (Fig. 1a) for the 139 type strains supported a rough separation of the family *Acetobacteraceae* into the acetous and acidophilic groups. The *Acetobacteraceae* genomes not classified as AAB (with the exception of *Acidocella aminolytica* 101^T^) showed a narrow GC content range (Δ ~ 11%mol) with values between 62.7%mol (*Roseomonas cervicalis* ATCC 49957^T^) and 73.9%mol (*Crenalkalicoccus roseus* YIM 78023^T^). However, this group showed a large variation in genome size (Δ ~ 4.8 Mbp), ranging from 3.03 Mbp (*Elioraea thermophila* YIM 72297^T^) to 7.78 Mbp (*Dankookia rubra* JCM 30602^T^). In contrast, the GC content of the AAB varied widely (Δ ~ 31%mol), with values between 36.8%mol (*Commensalibacter intestini* A911^T^) and 67.7%mol (*Endobacter medicaginis* LMG 26838^T^). However, this group showed less variation in genome size (Δ ~ 2.8 Mbp), ranging from 2.01 Mbp (“*Parasaccharibacter apium* A29^T^”) to 4.83 Mbp (*Gluconacetobacter sacchari* LMG 19747^T^). Interestingly, AAB genera isolated exclusively from the insect gut, consisting of the genera *Bombella* (= “*Parasaccharibacter*”), *Commensalibacter* and *Entomobacter*, clustered in a region of low genome size within the acetous group, suggesting an ongoing evolutionary reduction of genome size probably reflecting their symbiotic lifestyles.

### Phylogenomics

Phylogenomic analysis based on core protein sequences confirmed that the acetous group originated from a lineage, probably already inhabiting low-pH environments, derived from the more basal acidophilic group (Fig. 1b). *Acetobacteraceae* type strains were organized into suprageneric or infrageneric clades (Fig. 1b and Supplementary Table 2) according to the position in the phylogenomic tree. Nine distinct clades were recognized within the acidophilic group: the early separating branch containing the genus *Elioraea*, followed by a number of recently proposed groups reorganizing the genus *Roseomonas* (Rai et al. 2021), the pool of strains representing *Belnapia* and the related genera *Caldovatus*, *Crenalkalicoccus*, *Dankookia*, *Roseicella* and *Siccirubricoccus*, *Rhodovarius* and the related genera *Roseococcus* and *Rubritepida*, *Acidocella* and *Acidiphilium* strains, and finally the lineages composed by strains of *Acidibrevibacterium* and *Rhodovastum*, which shared a late common ancestor with the acetous group. The polyphyletic origin of the genus *Roseomonas* sensu stricto observed in this study is in agreement with the recent reclassification (Rai et al. 2021). The topology of the acetous group confirmed the current accepted demarcation of most genera with a few exceptions. As previously suggested (Yamada et al. 2012), the type strain *Gluconacetobacter entanii* LTH 4560^T^ belongs to the genus *Komagataeibacter*. Given the high-support nodes indicating late common ancestors within certain members of a same genus, we proposed subgroups (clades named according to the most ancient described type species) for the genera *Acetobacter*, *Gluconobacter* and *Komagataeibacter* (Fig. 1b). The topology of the phylogenomic tree obtained in this study is in full agreement with the latest accepted treatment (Hördt et al. 2020).

### Biosynthesis of specialized metabolites

**Fig. 2:**
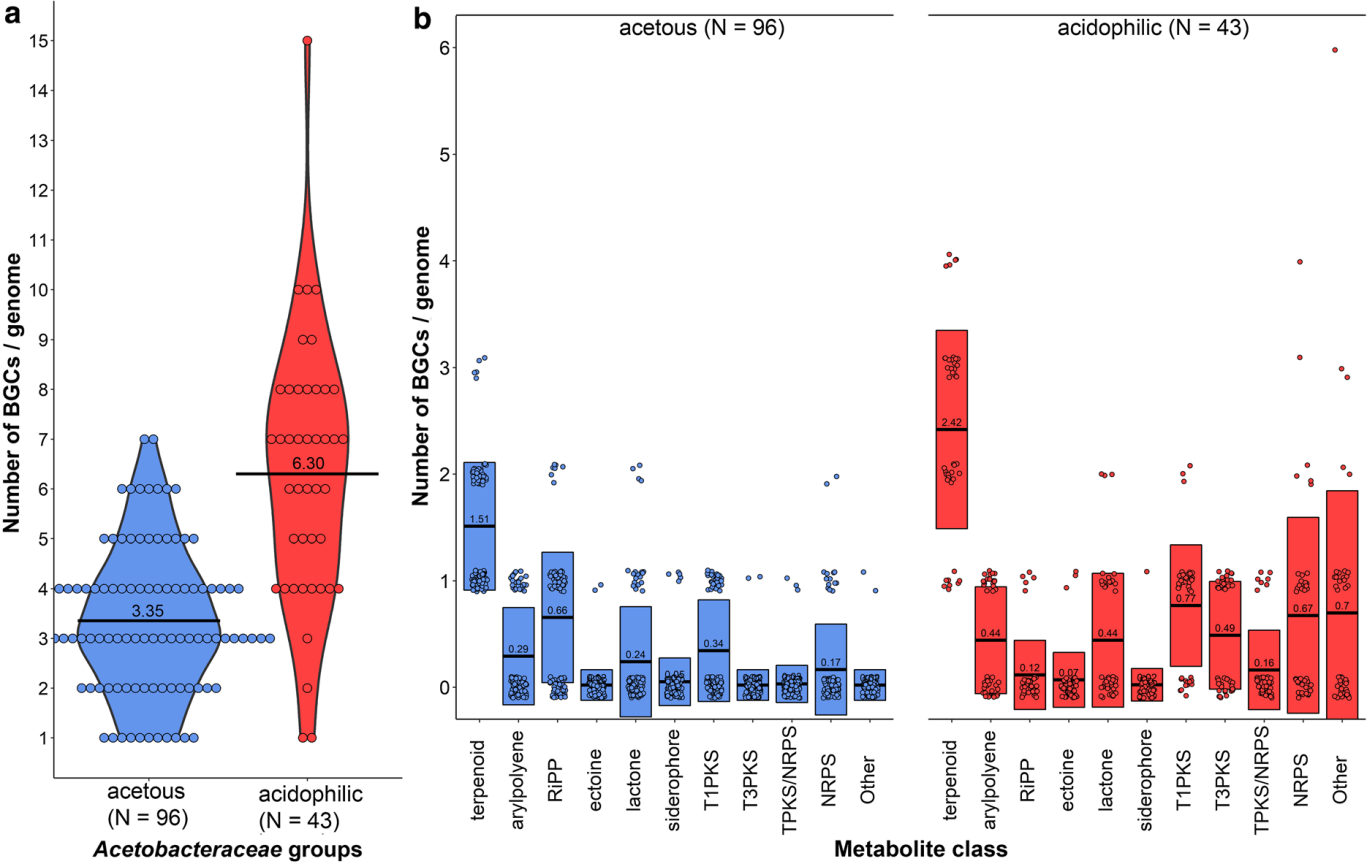
Presence of biosynthetic gene clusters (BGCs) in the two groups of the family *Acetobacteraceae*. **a** The number of BGCs per genome was plotted for each type strain, organized according to the taxonomic classification into acetous and acidophilic species. **b** BGCs for the biosynthesis of different metabolite classes were plotted for each type strain and were organized according to the taxonomic classification into acetous and acidophilic species. The numbers inside the boxplots are the calculated mean values

The BGCs identified in the family *Acetobacteraceae* using antiSMASH were organized in 10 groups according to the metabolite class or pathway: arylpolyene, ectoine, lactone, type-1 polyketide synthase (PKS), type-3 PKS, ribosomally synthesized and post-translationally modified peptide (RiPP), siderophore, terpenoid, non-ribosomal peptide synthetase (NRPS), NRPS/PKS hybrids, and miscellaneous BGCs. Because antiSMASH search is based on previously identified and studied clusters, it is possible that completely new biosynthetic pathways are missed, and thus the results here described do not necessarily exhibit the full biosynthetic potential of this group of understudied bacteria. All type strains of the family carried at least one BGC and the global average was 4.3 ± 2.3 BGCs/genome. Members of the acidophilic group carried on average 6.30 ± 2.63 BGCs/genome, which was twice as many as the acetous group, which carried on average 3.35 ± 1.48 BGCs/genome (Fig. 2a). This difference was statistically significant (*p* < 0.001) based on both the Kruskal–Wallis test and the Benjamini–Hochberg test. All the *Acetobacteraceae* type strains carried gene clusters involved in terpenoid biosynthesis. These BCGs were found on average at a frequency of ~ 2.4 BGCs/genome in the acidophilic group, and ~ 1.5 BGCs/genome in the acetous group (Fig. 2b). In general, the acidophilic group carried a larger number of class-specific BGCs than the acetous group, and this was particularly evident for PKS and NRPS genes (Fig. 2b). The genomes of some strains featured a high number of PKS-encoding genes, for example *Roseomonas stagni* DSM 19981^T^, whereas in other genomes, for example *Roseomonas aerophila* NBRC 108923^T^, NRPS-encoding genes were predominant. The one BGC that was present in higher numbers in the acetous group was involved in the production of RiPPs (Fig. 2b).

**Fig. 3:**
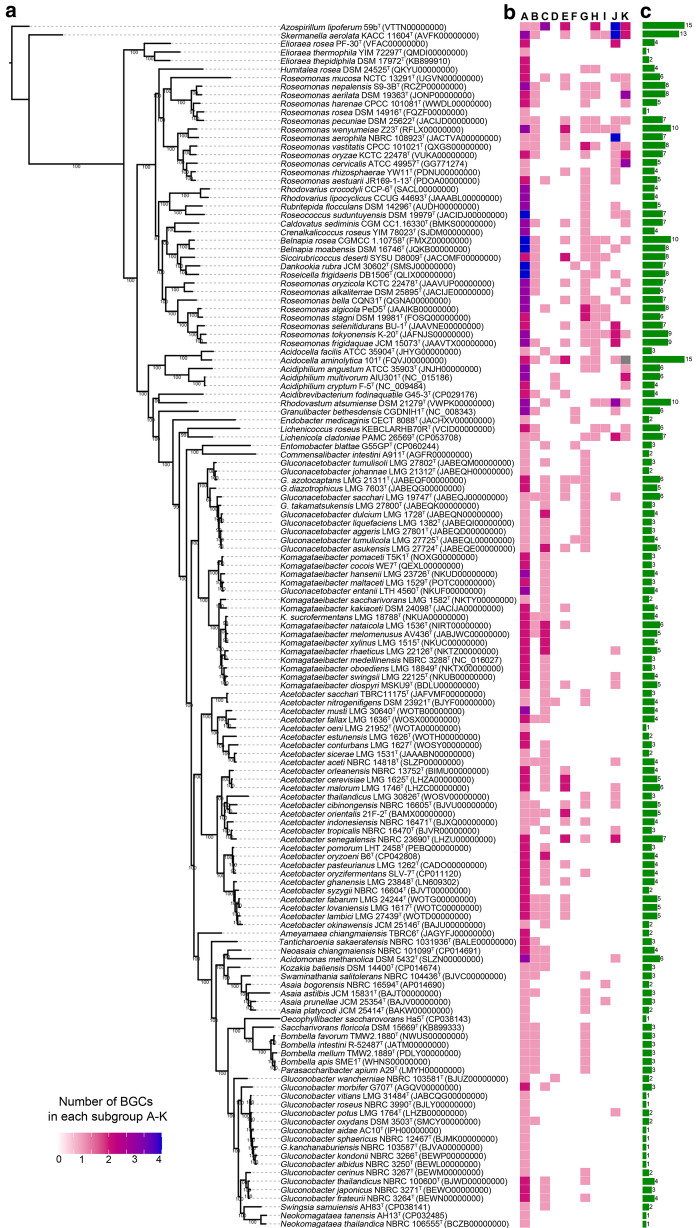
Phylogenomic analysis of the family *Acetobacteraceae* and their biosynthetic gene clusters (BGCs) as detected using antiSMASH. **a** Phylogenomic tree based on 50 housekeeping protein sequences. **b** Type and number of BCGs in the genomes of each type species. **c** Total number of BCGs with at least one core gene detected using antiSMASH. The subgroups were classified according to the class or pathway of the metabolite as follows: A = terpenoid, B = aryl polyene, C = ribosomally synthesized and post-translationally modified peptide, D = ectoine, E = lactone, F = siderophore, G = type-1 polyketide, H = type-3 polyketide, I = hybrid polyketide/non-ribosomal peptide, J = non-ribosomal peptide, K = other specialized metabolites

A direct correlation between taxonomy or phylogeny and the presence of certain types of BGCs was not evident, but certain trends were observed (Fig. 3). For example, the acidophilic group generally carried some miscellaneous gene clusters for phosphonates and indoles, whereas no acetous species carried these BGCs (Fig. 3ab). Most of the species (~ 81%) in the genus *Roseomonas* carried at least one polyketide cluster with the exception of *R. mucosa* NCTC 13292^T^, *R. rosea* DSM 14916^T^, *R. aerophila* NBRC 108923^T^ and *R. cervicalis* ATCC 49957^T^. All *Gluconacetobacter* species featured a type-1 PKS, whereas not a single strain of the related genus *Komagataeibacter* carried a polyketide cluster, and thus the product (still unknown) can be considered as a taxonomic marker. The genus *Acetobacter* tended to feature more BGCs among the acetous group (Fig. 3ac), with the highest numbers identified in the strain *A. senegalensis* LMG 23690^T^ (seven BGCs). *Acetobacter* species were proficient in the biosynthesis of lactones and non-ribosomal peptides, whereas members of the *Asaia-Bombella* clade carried gene clusters involved in the biosynthesis of polyketides.

### Aryl polyenes

Aryl polyenes (APEs) are bacterial pigments produced abundantly by the phylum *Proteobacteria*, and like carotenoids these unsaturated molecules play a role in the capture of free radicals to prevent oxidative stress (Schöner et al. 2016). The biosynthesis of APEs involves the loading of an aromatic precursor (usually 4-hydroxybenzoic acid) onto an acyl carrier protein (ACP), named ApeE, in a process catalyzed by ApeH (Grammbitter et al. 2020). The central enzyme β-ketoacyl-ACP synthase (ApeO/ApeC) elongates the chain in a decarboxylation Claisen condensation with malonate units, and the cetone product is reduced to alcohol by ApeQ and dehydrated to a double bond by ApI/ApeP in an iterative process. For some metabolites, the aryl polyene is linked to *N*-acetylglucosamine by the glycosyltransferase ApeJ. The presence of homologs of the core β-ketoacyl-ACP synthase among *Acetobacteraceae* allowed the identification of APE producers. The acidophilic group featured a higher proportion (19/43 = 44%) of APE gene clusters than the acetous group (28/96 = 29%), suggesting that bacteria readily exposed to sunlight, such as those inhabiting ponds, probably produce APEs for protection against UV radiation.

The β-ketoacyl-ACP synthase encoded in the genomes of *Gluconacetobacter sacchari* LMG 19747 T and *Swaminathania salitolerans* NBRC 104436^T^ differed from the other homologs in the family (Supplementary Fig. 1a), and were probably transferred horizontally from other organisms (likely from Gammaproteobacteria, given that homologous proteins were identified in *Yersinia, Serratia* and *Pseudomonas*). The unrooted tree of β-ketoacyl-ACP synthase (Supplementary Fig. 1a) was broadly congruent with the phylogenomic tree based on core genes (Fig. 1b), with a distinct separation of the acetous and acidophilic groups. Some genes linked to the β-ketoacyl-ACP synthase gene were tentatively annotated as encoding an adenylate-forming protein, a dehydrogenase, a probable halogenase, and transport proteins, whereas others were hypothetical. The aryl polyene BCGs of the family *Acetobacteraceae* appear likely to produce as yet undescribed aryl polyene products.

### Ectoines

Ectoines are bacterial natural products sharing a 4-carboxylic acid pyrimidine that promote survival in hyperosmotic environments (Czech et al. 2018). Ectoines are synthesized from l-aspartate-β-semialdehyde by the sequential action of three proteins: EctB, EctA and EctC (Czech et al. 2018). The final enzyme (EctC) is known as l-ectoine synthase, and catalyzes the transformation of *N*_4_-acetyl-l-2,4-diaminobutanoate to l-ectoine, acting as a marker for the identification of ectoine BGCs. Homologs of this EctC protein in *Paenibacillus lautus* NBRC 15380 (Czech et al. 2019) were detected in only five *Acetobacteraceae* type strains: *Acidocella aminolytica* 101^T^, *Acidiphilium cryptum* JF-5^T^, *Acidiphilium multivorum* AUI301^T^, *Acetobacter nitrogenificens* DSM 23291^T^ and *Gluconobacter wancherniae* NBRC 103581^T^ (Supplementary Fig. 1b). The three members of the acidophilic group have a full ectoine cluster, including genes encoding other enzymes in the pathway such as EctA, EctB, EctD and the l-aspartate kinase (Ask) together with a transporter and a transcriptional regulator. Interestingly, the acetous clusters with an *ectC* gene carried no linked homologs of *ectA*, *ectB, ectD* or *ask*, and thus it is uncertain whether the encoded EctC protein is a functional l-ectoine synthase or has a different role. An EctA homolog was identified in *A. nitrogenificens* DSM 23291^T^ but not in *G. wancherniae* NBRC 103581^T^. Homologs of the *N*-acetyltransferase EctB were detected in both strains but also in many other *Acetobacter* and *Gluconobacter* species, suggesting involvement in a more general pathway. Finally, no EctD homologs were detected in any species of the acetous group. These results suggest that the acetous group probably does not produce ectoines, and the functional role of EctC homologs in *A. nitrogenificens* DSM 23291^T^ and *G. wancherniae* NBRC 103581^T^ should be investigated in more detail.

### Hopanoids

The most common protein sequence encoded in the *Acetobacteraceae* terpenoid clusters was used as a blastp query, resulting in the identification of an enzyme involved in hopene biosynthesis. Hopanoids are pentacyclic bacterial triterpenoids that confer fluidity and integrity to the cell membrane in a similar manner to sterols (such as cholesterol and sitosterol) in eukaryotes (Sáenz et al. 2015). All *Acetobacteraceae* type strains carried genes for hopanoid synthesis. The most common hopanoid BGC consisted of genes for squalene-hopene cyclase (SHC) and two squalene synthases. This type of cluster was often found in the acetous group, and hopanoids may therefore protect the cell membrane against injury caused by acetic acid (Belin et al. 2018; Welander et al. 2009). In the acidophilic group, these biosynthetic genes were not clustered together and typically one or more was missing. SHC is the central enzyme of hopanoid biosynthesis and is responsible for the cascade polycyclization of squalene leading to the pentacyclic hopene triterpenoid (Siedenburg and Jendrossek 2011). The unrooted tree based on SHC amino acid sequences showed two groups (Supplementary Fig. 1c), the major group bearing a perceptible phylogenetic signal. Certain species of *Acetobacter*, *Gluconobacter* and *Komagataeibacter* encoded two versions of the SHC protein (Supplementary Fig. 1c), and although most *Acetobacteraceae* carried the most common SHC, some carried only the second type. This second SHC shared a consensus sequence of ~ 30 amino acids near the C-terminus that is not present in the major SHC or in homologous proteins from *Streptomyces* but is found in some species of the genera *Zymomonas*, *Bradyrhizobium* and *Rhodopseudomonas*. Homologs of the two versions of SHC found in certain *Acetobacteraceae* also occur in *Zymomonas mobilis* and their activity has been verified experimentally (Seitz et al. 2012). The three-gene hopanoid core BGC of the acetous group also contained additional genes for accessory proteins, the most common of which were annotated as a glycosyltransferase, a FAD-dependent oxidoreductase, and a NAD-dependent epimerase/hydratase. In a number of species of *Roseomonas* (such as *R. aerilata* DSM19363^T^ *R. nepalensis* S9^T^ and *R. oryzae* KCTC42542^T^), the core hopene cyclase gene was linked to a phosphoenolpyruvate mutase gene, which is the marker for organophosphonic acid synthesis (Horsman and Zechel 2017), suggesting it is part of a hybrid cluster that generates a yet unknown compound. In *Acetobacter malorum* LMG 1746^T^, the alternative hopene cyclase gene was linked to an auto-inducer synthase gene, indicating that further natural hopanoids with yet unknown functions may exist.

### Lactones

Two types of lactone BGCs were found in the *Acetobacteraceae* genomes, encoding the enzymes needed for the production of acyl-homoserine lactones (AHLs) and β-lactones, respectively. AHLs are involved in quorum sensing (QS), an intercellular communication process that triggers coordinated gene expression (Waters and Bassler 2005). AHLs are QS auto-inducing factors because they bind to a transcription factor (LuxR in *Aliivibrio fischerii*) which activates the expression of the gene encoding the AHL synthase (LuxI in *A. fischerii*), resulting in the massive production of AHLs throughout the population. LuxR and LuxI homologs are widespread in *Proteobacteria* (Case et al. 2008; Schuster et al. 2013). AHLs are produced from *S*-adenosylmethionine by cleavage, cyclization and *N*-acylation with an ACP or acyl-coenzyme A (Schaefer et al. 2018). Genes for AHL biosynthesis were identified in only three species of the acidophilic group (7%): *Acidocella aminolytica* 101^T^ (two clusters), *Acidibrevibacterium fodinaquatile* G45-3^T^ and *Roseomonas nepalensis* S9-3B^T^. However, they were found more frequently in the acetous group (19/96 = 20%), being present in four species of *Gluconacetobacter*, three species of *Komagataeibacter* and twelve species of *Acetobacter*. Detailed analysis of protein alignments of the auto-inducer synthases (LuxI homologs) revealed three major groups, two of them specific for *Acetobacter*, and the third shared between *Komagataeibacter* and *Gluconacetobacter* (Supplementary Fig. 1d). Some *Acetobacter* species belonging to the orleanensis clades (*A. cerevisiae* LMG 1625^T^, *A. malorum* LMG 1746^T^ and *A. orientalis* 21F-2^T^) carried both types of auto-inducer synthases, suggesting the importance of QS in certain AABs used for the fermentation of must, fruit and cereal (Guillamón and Mas 2009; Iida et al. 2008; Valera et al. 2016). The protein sequence of the auto-inducer synthases from the acidophilic group were distantly related to those identified in the acetous group. Particularly those found in *A. fodinaquatile* G45-3^T^ and *R. nepalensis* S9-3B^T^ showed to have significantly different sequences, evidenced by the long branches in the phylogenomic tree (Supplementary Fig. 1d), suggesting the existence of an alternative QS mechanism (or a different biochemical function) that should be investigated in future experiments.

BGCs responsible for β-lactone biosynthesis were not identified in the acetous group but were found in thirteen acidophilic type strains, exclusive of the *Roseomonas* and *Belnapia* clades. The species *Roseomonas wenyumeiae* Z23^T^ and *Siccirubricoccus deserti* SYSU D8009^T^ carried two versions of the β-lactone BGC. Three β-lactone core enzymes were encoded by β-lactone clusters: a β-lactone AMP-binding protein supposedly catalyzing the coupling of a carboxylic acid (such as acetate) to coenzyme A, an HGML-like protein catalyzing the Claisen condensation of the acyl-CoA with a carboxylic acid to produce a β-ketoacid, and a dehydrogenase that reduces the intermediate to a β-hydroxyacid (Robinson et al. 2019). The final cyclization to the β-lactone is carried out by an ATP-dependent cyclase homologous to OleC (Robinson et al. 2019), but such a protein was not encoded by any of the BGCs. It is therefore unclear whether the product of these clusters is a β-lactone or a β-hydroxyacid. The β-hydroxyacid product may be a precursor in another specialized pathway, given that the β-lactone cluster in some *Roseomonas* strains (such as *R. pecuniae* DSM 25622^T^ and *R. vastitatis* CPCC 1121^T^) is fused with an NRPS cluster.

### Polyketides

We identified type-1 and type-3 PKS genes in the *Acetobacteraceae* type strains. Type-1 PKS genes encode large proteins organized into modules that use ACPs to activate acyl-CoA substrates, whereas type-3 PKS genes encode products that act directly on acyl-CoA substrates and often produce cyclized aromatic polyketides (Jenke-Kodama et al. 2005; Shen 2003). We detected a type-1 PKS in 70% of the acidophilic species (30/43) and in around one third of the acetous species (33/96). The PKS genes were found in specific taxonomic groups such as the *Roseomonas* clade (Fig. 3ab), both clades of *Gluconacetobacter*, as well as *Asaia* and *Bombella-Saccharibacter* and in certain species of *Acetobacter* and *Gluconobacter*. For a yet unknown reason, type-1 PKS genes were not found in the genus *Komagataeibacter*. The high degree of PKS conservation in the different AAB clades rules out horizontal transfer and suggests that the resulting metabolites conferred advantages on the common ancestor and remain beneficial to the extant species in their current ecological context.

**Fig. 4:**
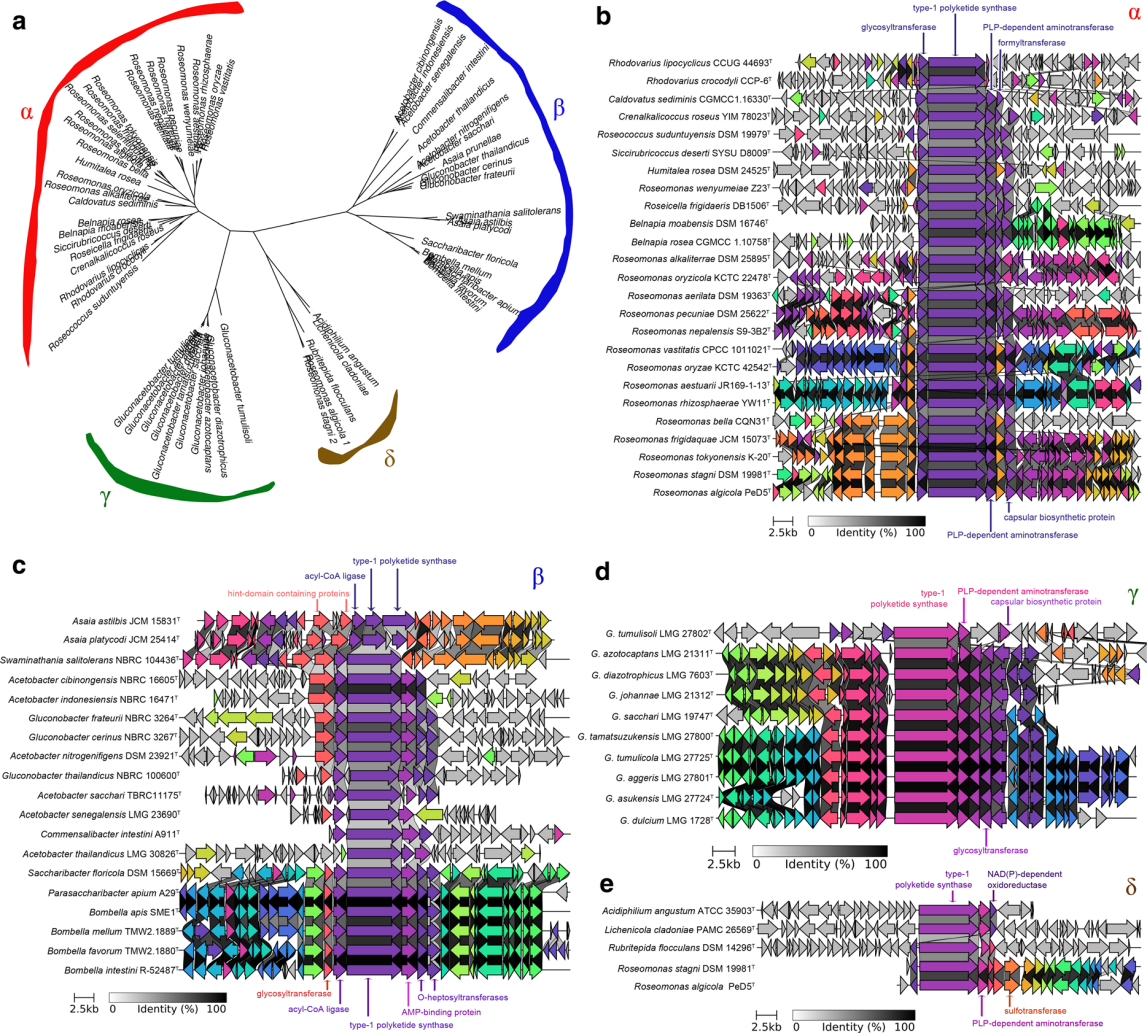
Type-1 polyketide synthase biosynthetic gene cluster in *Acetobacteraceae*. **a** Unrooted tree based on type-1 PKS showing the differentiation into four groups labelled α, β, γ and δ which correlate with certain taxonomic clades. Organization of the biosynthetic gene clusters for the type-1 PKS from the groups α **b** β **c** γ **d** and δ **e** showing the probable annotation of certain genes according to antiSMASH and blast analysis

A basic motif found in most *Acetobacteraceae* type-1 PKS proteins consisted of the ordered domains KS-AT-DH-ER-KR-PP (ketosynthase-acyltransferase-dehydratase-enoylreductase-ketoreductase-phosphopantheteine acyl carrier). In each strain, this basic motif was accompanied by a variety of small domains including aminotransferases (AmT), AMP-binding domains (A), coenzyme A-binding domains (CAL), enoyl-CoA hydratase/isomerase domains (ACH), NAD-dependent epimerase/dehydratase domains (NAD), further KR or ER domains, and/or a combination of such domains. Intriguingly thioesterase domains could not be identified within the PKS protein or as stand-alone accessory proteins. In all cases, the KS domains clustered with the type-1 PKS gene, such as those associated with the synthesis of aureothin or certain aromatic polyketides (Chen and Du 2016). The presence of a single module suggests that the *Acetobacteraceae* PKS system is iterative and not modular. The PKS amino acid sequence is considered a good proxy to infer the number of metabolic products. The unrooted tree of *Acetobacteraceae* type-1 PKS proteins based on sequence alignment revealed four different clades, which we named α, β, γ and δ (Fig. 4a). The α-group included all PKS proteins from the *Roseomonas* clade, except a second type-1 PKS identified in *Roseomonas stagnii* DSM 19981^T^ and *Roseomonas algicola* PeD5^T^, both of which clustered in the δ-group together with *Acidiphilium angustum* ATCC 35903^T^, *Lichenicola cladoniae* PAMC 26569^T^ and *Rubritepida flocculans* DSM 14296^T^. The PKS of the α-group carried phylogenetic signal as the clades *Pararoseomonas*, *Pseudoroseomonas*, *Belnapia*, *Neoroseomonas* and Falsiroseomonas were clearly distinguished (Fig. 4a). In all the α-group, a glycosyltransferase gene probably belonging to family GT4 (Breton et al. 2005), was found upstream of the PKS gene (Fig. 4b). The α-group also included genes for a PLP-dependent aminotransferase, a formyltransferase and a capsular biosynthetic protein. The metabolite produced by these α-group type-1 PKS clusters is anticipated to have the same skeleton decorated with small variations given the different accessory proteins encoded within each cluster. The β-group included PKS proteins from the genera *Acetobacter* and *Gluconobacter*, and a branching group leading to the *Asaia* and *Bombella* clades (Fig. 4a). The β-group type-1 PKS clusters differed from α, γ and δ clusters given the absence of a PLP-dependent aminotransferase gene, and instead the PKS gene was flanked by acyl ligase genes (Fig. 4c). The PKS-encoding gene in *Asaia* spp. likely split into two genes, and is accompanied by proteins having hint-domain. *O*-heptosyltransferase gene was also consistently found in the β-group clusters, and was duplicated in the *Bombella* clade and the *Gluconobacter* spp. clusters. In addition, the PKS cluster from the *Bombella* clade was closely related to the cluster found in *Swaminathania salitolerans* NBRC 104436^T^ and both clusters shared the presence of an additional glycosyltransferase and a thioredoxin, suggesting they may produce sulfur-containing metabolites. The γ-group was restricted to members of the genus *Gluconacetobacter*, and intriguingly these PKS proteins were more closely related to those from the acidophilic group rather than the rest of the acetous group (Fig. 4a) The γ-group type-1 PKS clusters (Fig. 4d) were highly conserved in gene organization and protein sequence, and probably synthesize the same metabolite, perhaps with the exception of *G. tumulisoli* LMG 27802^T^. These clusters encoded a PLP-dependent aminotransferase, two capsular biosynthetic proteins and two glycosyltransferases. A small number of type-1 PKS from acidophilic species clustered in the δ-group (Fig. 4a), and the BGCs (Fig. 4e) encoded a PLP-dependent aminotransferase and an oxidoreductase (and a sulfotransferase for the BGCs in *Roseomonas* spp.), located near the central type-1 PKS gene.

A type-3 PKS was identified in 21 of the 43 acidophilic type strains (49%) and from the acetous species, only in the lichenous strains *Lichenicoccus roseus* KEBCLARHB70R^T^ and *Lichenicola cladoniae* PAMC 26569^T^. This correlates with a specific evolutionary niche within plants but not lichens, where the metabolic product of the type-3 PKS cluster was unnecessary for phytosphere adaptation. Type-3 PKS was found in several strains of the genera *Roseomonas* (11/21 = 52%). The closest sequences beyond the *Acetobacteraceae* were identified in other *Alphaproteobacteria*, including *Azospirillium*, *Methylopila*, *Microvirga* and *Paracoccus* species. A similar type-3 PKS is ArsC (sequence identity ~ 29%) from *Azotobacter vinelandii* strain OP, which produces alkylresorcinols and alkylpyrones to protect its cysts against environmental injury (Funa et al. 2006). The type-3 PKS proteins from the acidophilic group are therefore likely to be involved in pyrone or resorcinol biosynthesis, and may also play a protective role because this group of bacteria thrives in sediments, soils, ponds and hot springs (Komagata et al. 2014) where solar radiation and desiccation can be detrimental. Type-3 PKS proteins from the family *Acetobacteraceae* could be assigned to three groups based on sequence alignment and phylogeny (Supplementary Fig. 2a). Specifically, we observed the divergence of *Roseomonas frigidaquae* JCM 15073^T^ and *Roseomonas stagni* DSM 19981^T^ (closer to *Belnapia* and *Siccirubricoccus* than to the main *Roseomonas* group). In addition to the central type-3 PKS, two other proteins were encoded in all the clusters: a methyltransferase and a FAD-dependent monooxygenase (Supplementary Fig. 2b). Interestingly, more closely related homologs of the methyltransferase were identified in other *Rhodospirillales*, such as *Azospirillium*, *Indioceanicola* and *Skermanella* species, but also in the myxobacterium *Sorangium cellulosum*, a recognized producer of specialized metabolites (Schneiker et al. 2007). The presence of methyltransferases and flavin-dependent monoxygenases is a common feature of certain type-3 PKS clusters particularly those found in fungi (Navarro-Muñoz and Collemare 2020) and in some myxobacteria (Hug et al. 2019), but the metabolite produced by *Rhodospirillales* is currently unknown.

### NRPS and hybrid NRPS/PKS clusters

NRPS genes were present in 47% (20/43) of the acidophilic species, and some strains featured multiple NRPS or NRPS-like clusters such as *R. aerophila* NBRC 108923^ T^ with four. The NRPS clusters were much less common among the acetous species, being present in only 15% (14/96). Like PKS genes, NPRS genes encode megasynthases organized into modules, including condensation (C), adenylation (A), thiolation (also known as peptidyl carrier protein, PCP), and thioesterase (TE) domains. Like the ACP in PKS, the PCP is activated by the transfer of a 4′-phosphopantetheine factor. Among the acidophilic species, seven of the 28 NRPS genes were trimodular, five were monomodular, six were bimodular, six were tetramodular, two were pentamodular and one hexamodular and one octamodular (*Roseomonas wenyumeiae* Z23^T^). In contrast, ten of the 15 NRPS genes in the acetous group were monomodular, two were bimodular, one trimodular, one pentamodular and one hexamodular. The lower number of NRPS clusters among the acetous species may probably reflect genome reduction induced by plant speciation events. The lack of these specific NRPS clusters in both *Acetobacteraceae* clades exclusive to insects (Bonilla-Rosso et al. 2019) is consistent with this hypothesis, and suggests that such peptides are probably more important for bacteria living in soil, sediment or water environments, where there exists higher chances of encountering diverse microbes.

Only a few species carried complete C-A-PCP-TE domains in a single protein (Supplementary Fig. 2c). In the acetous group, only two closely related *Acetobacter* species (*A. malorum* LMG 1746^T^ and *A. cibinongensis* NBRC 16605^T^) featured these domains in a single monomodular NRPS, whereas *A. aceti* NBRC 14818^T^ featured NPRS genes with the C-A-PCP-TE domains split into adjacent modules and also contained further modules with AmT and CAL domains, which are more common in PKS genes. Two strains of *Komagataeibacter* carried an NRPS-like cluster (*K. diospyri* MSKU9^T^ and *K. swingsii* LMG 22125^T^), but in both cases the NRPS gene contained A, PCP and TE domains, but no apparent C domain, suggesting either that C domains are provided by non-canonical hypothetical genes or that the cluster does not express a functional NRPS product and may be involved in other biosynthesis reactions, or maybe it is the result of translocation or recombination events. Similar NRPS-like genes encoding A, PCP and TE but not C domains were found in *Roseomonas vastitatis* CPCC 101021^T^ but its organization and the composition of accessory genes was different. Complete C-A-PCP-TE domains in a single module were also observed in trimodular clusters from *Roseomonas frigidaquae* JCM 15073^T^ and *Rhodovastum atsumiense* DSM 21279^T^, in tetramodular clusters from *Roseomonas rhizosphaerae* YW11^T^ and *Roseomonas rosea* DSM 14916^T^, in a pentamodular cluster from *Roseomonas aestuarii* JR169-1-13^T^, and in a hexamodular cluster from *Roseomonas mucosa* NCTC 13291^T^. The accompanying modules may provide adenylation or AMT, KR and ECH domains, which are most often found in PKS clusters, or a combination of these. Most of the NRPS clusters from the acidophilic group did not possess the complete minimal set of C-A-PCP-TE domains, and it is unclear if functional peptides are produced by these clusters. It is possible that non-canonical NRPS domains remain undetected by the current algorithm and are hidden in hypothetical accessory proteins. Multimodular NRPS clusters with repetitive C-A and KR domains, respectively, were identified in *Acetobacter senegalensis* LMG 23690^T^ and *Komagataeibacter rhaeticus* LMG 22126^T^.

Chimeric or hybrid NRPS-PKS clusters with contiguous PKS and NRPS modules were identified in *Belnapia rosea* CGMCC 1.10758^T^, *Lichenicoccus roseus* KEBCLARHB70R^T^, *Roseomonas algicola* PeD5^T^, *Roseomonas stagni* DSM 19981^T^, *Roseomonas tokyonensis* K-20^T^, *Roseomonas wenyumeia* Z23^T^ and *Siccirubricoccus deserti* SYSU D8009^T^. The hybrid cluster of *Roseomonas algicola* PeD5^T^ showed a complex architecture, showcasing fourteen modules, containing two NRPS modules flanked by seven PKS modules and a number of accessory domains. The presence of two genes encoding for different efflux proteins within the BGC, suggest that the produced metabolite is biologically active, and merit exploration. Hybrid clusters present in *Asaia bogorensis* NBRC 16594^T^ and *Asaia astilbis* JCM 15831^T^ encoded two megasynthases (one PKS and one NRPS) in opposing reading directions. Gene expression in these clusters is probably regulated by a histidine kinase receptor. The NRPS amino acid sequence showed some similarity to vicibactin VbsS from *Rhizobium* spp. (Heemstra et al. 2009), and this megasynthase may similarly catalyze the trimerization of certain amino acid residues. These hybrid clusters are unique among the *Acetobacteraceae* type strains and they are likely to produce undiscovered bioactive metabolites, which deserve further detailed study.

### Ribosomally synthetized and post-translationally modified peptides

RiPP gene clusters were identified in a handful of acidophilic strains including *Acidocella facilis* ATCC 35904^T^, *Rhodovastum atsumiense* DSM 21279, *Roseomonas algicola* PeD5^T^, *Roseomonas aestuarii* JR169-1-13^T^ and *Roseomonas mucosa* NCTC 13291^T^. In contrast, such clusters were much more prevalent in the acetous group (Fig. 2b), being present in all *Komagataeibacter* strains, all *Gluconacetobacter* strains except *Gluconacetobacter johannae* LMG 21312^T^, in 75% (21/28) of the *Acetobacter* strains, and in 27% (4/15) of *Gluconobacter* strains (Fig. 3ab). Interestingly, no RiPP clusters were found in *Asaia* or in *Saccharibacter-Bombella* clades. The only insect-associated AAB type strain carrying a RiPP cluster was *Entomobacter blattae* G55GP^T^, which is predicted to produce a yet unknown linear azol(in)e peptide. *Roseomonas mucosa* NCTC 13291^T^ was the only species to also carry a BGC encoding a YcaO cyclohydratase, which catalyzes ring formation in azol(in)e peptides. *Roseomonas algicola* PeD5^T^ was the only species of the family to be predicted to produce a lasso peptide. Finally, a cyanobactin peptidase gene involved in the final step of RiPP maturation was found in *Roseomonas oryzae* KCTC 42542^T^, and it is likely that this strain produces a new cyanobactin-like peptide.

The RiPP clusters found in the acidophilic group (except *Roseomonas mucosa* NCTC 13291^T^) encoded a DUF692-like protein homologous to MbnB from *Methylosinus trichosporium* OB3b, which binds iron and forms a complex with MnbC to catalyze the formation of an oxazolone-thioamide group on the core peptide sequence of methanobactin, a copper-chelating molecule (Kenney et al. 2018). In those species with a DUF692-like cluster, we did not identify a leader sequence or homologs of MnbC or the TonB receptor. However, this leader-core peptide sequence along with MnbC and TonB homologs were identified in the acetous group. Accordingly, *Acetobacter oryzoeni* B6^T^, *Gluconacetobacter asukensis* LMG 27724^T^, *Komagataeibacter nataicola* LMG 1536^T^, *K. rhaeticus* LMG 22126^T^ and *K. xylinus* LMG 1515^T^ are likely to produce as yet uncharacterized molecules related to methanobactins. The core DUF692 protein encoded by *A. oryzoeni* B6^T^, *K. rhaeticus* LMG 22126^T^ and *K. xylinus* LMG 1515^T^ had exactly the same sequence. An unrooted tree based on the DUF692 protein agreed well with the existing phylogeny, clearly distinguishing the acetous and acidophilic groups (Supplementary Fig. 2d), and intriguingly the protein from *G. asukensis* LMG 27724^T^ was located in the acidophilic cluster.

The RiPP clusters found in acetous species can be assigned to two major groups: the DUF692 cluster also found in the acidophilic species and the linocin M18 cluster. The latter was exclusive to acetous species and was the most common cluster after the hopanoids, being present in 55/96 (~ 57%) of the acetous type strains. This cluster was present in all type strains of the genus *Komagataeibacter*, in 10/11 (91%) of the *Gluconacetobacter* and in (21/28) 75% of the *Acetobacter* species. *Gluconacetobacter dulcium* LMG 1728^T^ and *Gluconacetobacter tumulisoli* LMG 27802^T^ featured two linocin M18 clusters, and *Gluconacetobacter aggeris* LMG 27801^T^ and *Gluconacetobacter tumulicola* LMG 27725^T^ shared exactly the same core linocin M18 protein sequence. Intriguingly, the linocin M18 cluster was not found in any *Asaia* or *Neokomagataea* species, or in the *Bombella-Saccharibacter* and *Ameyamaea-Tanticharoenia* clades, suggesting this pathway is required for certain yet unknown ecological relationships with plants. The unrooted tree based on the linocin M18 protein (Supplementary Fig. 2e) was interesting because there was no clear genus demarcation between *Acetobacter*, *Komagataeibacter* and *Gluconacetobacter*. This suggests either evolution from a common ancestor with independence from constraints operating on core phylogenetic-signal carrying genes, or horizontal gene transfer. Notably, this cluster was not present in any of the insect-associated clades. Because none of the basal acidophilic strains can produce the linocin M18 biosynthetic protein, the ancestor protein in AAB was probably transferred from plant-dwelling members of the family *Nitrobacteraceae* such as *Bradyrhizobium*, given the presence of homologs in this genus. A similar linocin M18 cluster has been studied in *Rhodococcus jostii* RHA1 and was found to encode a DypB peroxidase and an encapsulin protein that together generate a biochemically active lignin degradation nano-compartment (Rahmanpour and Bugg 2013). The linocin cluster found in AAB also encoded an encapsulin and a Dyp-type peroxidase, suggesting this cluster is involved in lignin degradation.

### Siderophores

Siderophores are iron-scavenging metabolites that allow producers to thrive in iron-depleted environments. They are particularly useful for microbial competition and are considered virulence factors in pathogenic organisms (Miethke and Marahiel 2007). Only NRPS-independent pathways for siderophore biosynthesis (Oves-Costales et al. 2009) were identified in the *Acetobacteraceae*, particularly in the basal phylogenetic clades of the acetous group (5/94) and only in one strain of the acidophilic group, *Dankookia rubra* JCM 30602^T^ (Fig. 3a, b). Two types of siderophore BGCs were identified. One cluster, shared by *D. rubra* JCM 362^T^ and *Granulibacter bethesdensis* CGDNIH1^T^, encoded two NRPS-independent siderophore synthases (IucA/IucC-like) (Supplementary Fig. 2f), homologous to proteins from strains of the order *Hyphomycrobiales* (class *Alphaproteobacteria*) such as *Methylobacterium*, *Pseudovibrio* and *Brucella* spp. In addition, the cluster encoded an *N*-acetyltransferase and a flavin-dependent lysine *N*-monooxygenase, and the resulting metabolite is probably a yet undescribed siderophore. The second cluster was shared by two *Gluconacetobacter* species (*G. azotocaptans* LMG 21311^ T^ and *G. tumulicola* LMG 27725^ T^) and encoded a single IucA/IucC-like synthase and for a number of proteins of unknown function (Supplementary Fig. 2 g). The siderophore cluster of *E. blattae* G55GP^T^ is unique in the family *Acetobacteraceae* and the encoded proteins show homology to proteins from strains of the genera *Azotobacter* and *Pseudomonas*, and are distantly related to the clusters for vibrioferrin and xanthoferrin biosynthesis (Pandey et al. 2017; Tanabe et al. 2003). The siderophore cluster identified in the genome of *Endobacter medicaginis* LMG 26383^T^ is unique within the family and includes next to the *iucA/iucC* marker, a gene encoding for an anthranilate isomerase, a reaction typical of the phenazine biosynthetic pathway.

### Miscellaneous biosynthetic clusters

The acidophilic type strains also encoded biosynthetic proteins for less common specialized metabolites such as phosphonates and indoles, but such clusters were not present in the acetous species. Thirteen strains (27%) in the acidophilic group encoded a homolog of phosphoenolpyruvate mutase and are likely to produce uncharacterized phosphonates. The presence of pyruvate decarboxylase and aminotransferase genes adjacent to the mutase indicated the formation of phosphonoacetaldehyde and finally 2-aminoethylphosphonate, which may be integrated into variety of end-products (Horsman and Zechel 2017). Two classes of phosphoryl mutase were identified in the clade, a shorter version present in *Rhodovastum atsumiense* DSM 21279^ T^ and *Roseomonas oryzicola* KCTC 22478^T^, and the larger and most frequent version in *Belnapia rosea* CGMCC 1.10758^T^ and five *Roseomonas* strains (Supplementary Fig. 2 h). Terpenoid biosynthesis genes were often closely linked to the mutase gene, suggesting that the product is an undiscovered terpene-phosphonate. *N*-acyl amino acids are synthesized from corresponding amino acid precursors by homologs of the *N*-acyl amino acid synthase NasY (Craig et al. 2011). Interestingly, NasY homologs were found exclusively in three type strains of the genus *Acidiphilium*, and this biosynthetic property is likely to be a marker of this genus. A putative homolog of PhzB, which catalyzes the synthesis of phenazine, was identified in the genome of *R. vastitatis* CPCC 101021^T^, but no other genes related to phenazine biosynthesis were found in the vicinity. PhzB is a member of the nuclear transport factor 2 (NTF2) family, which may have other functions in bacterial cells (Eberhardt et al. 2013), so it is not yet clear whether this strain can produce phenazines.

## Conclusion

The family *Acetobacteraceae* belongs to the class *Alphaproteobacteria*, and members of this class are not generally considered prolific producers of specialized metabolites, despite some strains carrying more than forty BGCs (Mukherjee et al. 2017). A relatively small number of molecules have been characterized from this taxonomic class, however so far, no specialized metabolites (< 2 kDa) have been purified from strains of the family *Acetobacteraceae*. In this study we were able to predict that all members of the *Acetobacteraceae* are producers of hopanoids. These triterpenoids play a fundamental role in the integrity of the bacterial cell membrane, particularly under stressful conditions such as low pH, but given the presence of two distinct hopanoid BGCs in a number of acetous species it is possible that these metabolites have additional functions. The acidophilic group featured almost twice as many BGCs as the acetous group. Most of the strains in both groups carried at least one type-1 PKS, and most members of the acidophilic group showed at least one NRPS and one type-3 PKS. The acetous group was found to produce ribosomally synthetized peptides belonging to the linocin M18—encapsulin family. A smaller number of strains in both groups appear able to produce aryl polyenes, lactones and siderophores. Thus far, none of these specialized metabolites have been purified, and the translation of metabolic potential in silico to actual metabolic capability remains to be confirmed. Given the diverse ecological niches occupied by the *Acetobacteraceae*, including ponds, sludge, soil, sediments, fruits, flowers and insect guts, the specialized metabolites produced by these species are likely to be bioactive and may be suitable for biotechnological exploitation.

## Supplementary Information

Below is the link to the electronic supplementary material.

Supplementary file1 (DOCX 1619 kb)

## Data Availability

Datasets generated during this study are available to interested readers on request.
