# Supplementary material for: Genome analysis suggests the bacterial family Acetobacteraceae is a source of undiscovered specialized metabolites
Source: Antonie Van Leeuwenhoek. 2021 Nov 10;115(1):41–58. doi: 10.1007/s10482-021-01676-7 (PMC8776678; doi:10.1007/s10482-021-01676-7)
Supplement: Supplementary file 1 — Supplementary file1 (DOCX 1619 kb) [file 10482_2021_1676_MOESM1_ESM.docx]

**Antonie van Leeuwenhoek**

Supplementary Information

**Genome analysis suggests the bacterial family *Acetobacteraceae* is a source of undiscovered specialized metabolites**

Juan Guzman^1,*^, Andreas Vilcinskas^1,2^

1 Department of Bioresources, Fraunhofer Institute for Molecular Biology and Applied Ecology, Ohlebergsweg 12, D-35392 Giessen, Germany

2 Institute for Insect Biotechnology, Justus-Liebig-University of Giessen, Heinrich-Buff-Ring 26-32, D-35392, Giessen, Germany

Corresponding author:

Juan Guzman

juan.guzman@ime.fraunhofer.de

Telefon +49 641 9937761

Fax +49 641 4808581

ORCID:

Juan Guzman (0000-0003-4120-9065)

Andreas Vilcinskas (0000-0001-8276-4968)

**Supplementary Table 1. Type strains with published genomes used in this study.**

| **Species and strain** | **Accession** | **No. contigs** | **N50 (bp)** | **Size (Mbp)** | **G+C content (%)** |
| --- | --- | --- | --- | --- | --- |
| *Acetobacter aceti* NBRC 14818^T^ | SLZP00000000 | 57 | 144080 | 3.63 | 57.1 |
| *Acetobacter cerevisiae* LMG 1625^T^ | LHZA00000000 | 157 | 51728 | 3.09 | 58.0 |
| *Acetobacter cibinongensis* NBRC 16605^T^ | BJVU00000000 | 40 | 180151 | 3.10 | 54.4 |
| *Acetobacter conturbans* LMG 1627^T^ | WOSY00000000 | 88 | 230861 | 3.88 | 59.0 |
| *Acetobacter estunensis* LMG 1626^T^ | WOTH00000000 | 120 | 75687 | 3.45 | 59.7 |
| *Acetobacter fabarum* LMG 24244^T^ | WOTG00000000 | 68 | 192685 | 3.08 | 57.8 |
| *Acetobacter fallax* LMG 1636^T^ | WOSX00000000 | 163 | 88078 | 4.22 | 58.3 |
| *Acetobacter ghanensis* LMG 23848^T^ | LN609302 | 3 | 2664884 | 2.84 | 56.9 |
| *Acetobacter indonesiensis* NBRC 16471^T^ | BJXQ00000000 | 77 | 180498 | 3.40 | 53.9 |
| *Acetobacter lambici* LMG 27439^T^ | WOTD00000000 | 193 | 75608 | 3.25 | 57.2 |
| *Acetobacter lovaniensis* LMG 1617^T^ | WOTC00000000 | 71 | 189100 | 3.47 | 57.6 |
| *Acetobacter malorum* LMG 1746^T^ | LHZC00000000 | 57 | 137929 | 3.83 | 56.7 |
| *Acetobacter musti* LMG 30640^T^ | WOTB00000000 | 138 | 141422 | 4.71 | 59.5 |
| *Acetobacter nitrogenifigens* DSM 23921^T^ | BJYF00000000 | 113 | 174239 | 4.24 | 60.5 |
| *Acetobacter oeni* LMG 21952^T^ | WOTA00000000 | 111 | 120458 | 4.11 | 58.5 |
| *Acetobacter okinawensis* JCM 25146^T^ | BAJU00000000 | 127 | 66162 | 3.17 | 57.6 |
| *Acetobacter orientalis* 21F-2^T^ | BAMX00000000 | 66 | 159374 | 3.11 | 52.4 |
| *Acetobacter orleanensis* NBRC 13752^T^ | BJMU00000000 | 78 | 151988 | 3.00 | 56.5 |
| *Acetobacter oryzifermentans* SLV-7^T^ | CP011120 | 4 | 2799488 | 3.11 | 52.4 |
| *Acetobacter oryzoeni* B6^T^ | CP042808 | 4 | 2744393 | 3.15 | 53.1 |
| *Acetobacter pasteurianus* LMG 1262^T^ | CADO00000000 | 141 | 90758 | 2.98 | 53.1 |
| *Acetobacter pomorum* LHT 2458^T^ | PEBQ00000000 | 212 | 75942 | 3.33 | 51.7 |
| *Acetobacter sacchari* TBRC11175^T^ | JAFVMF00000000 | 60 | 195911 | 4.55 | 60.1 |
| *Acetobacter senegalensis* LMG 23690^T^ | LHZU00000000 | 148 | 64691 | 3.93 | 55.6 |
| *Acetobacter sicerae* LMG 1531^T^ | JAAABN000000000 | 93 | 248749 | 4.03 | 57.6 |
| *Acetobacter syzygii* NBRC 16604^T^ | BJVT00000000 | 43 | 174631 | 2.68 | 55.5 |
| *Acetobacter thailandicus* LMG 30826^T^ | WOSV00000000 | 45 | 411907 | 2.80 | 50.5 |
| *Acetobacter tropicalis* NBRC 16470^T^ | BJVR00000000 | 186 | 93176 | 3.53 | 55.8 |
| *Acidibrevibacterium fodinaquatile* G45-3^T^ | CP029176 | 4 | 3907406 | 4.04 | 65.6 |
| *Acidiphilium angustum* ATCC 35903^T^ | JNJH00000000 | 206 | 71968 | 4.18 | 63.6 |
| *Acidiphilium cryptum* F-5^T^ | NC_009484 | 9 | 3389227 | 3.96 | 67.1 |
| *Acidiphilium multivorum* AIU301^T^ | NC_015186 | 9 | 3749411 | 4.21 | 67.0 |
| *Acidocella aminolytica* 101^T^ | FQVJ00000000 | 109 | 84959 | 3.96 | 58.9 |
| *Acidocella facilis* ATCC 35904^T^ | JHYG00000000 | 48 | 286929 | 3.40 | 64.5 |
| *Acidomonas methanolica* DSM 5432^T^ | SLZN00000000 | 96 | 124026 | 3.68 | 64.7 |
| *Ameyamaea chiangmaiensis* TBRC6^T^ | JAGYFJ000000000 | 36 | 358580 | 3.40 | 64.8 |
| *Asaia astilbis* JCM 15831^T^ | BAJT00000000 | 49 | 146992 | 3.15 | 58.0 |
| *Asaia bogorensis* NBRC 16594^T^ | AP014690 | 1 | 3198265 | 3.20 | 59.8 |
| *Asaia platycodi* JCM 25414^T^ | BAKW00000000 | 35 | 117874 | 3.15 | 59.3 |
| *Asaia prunellae* JCM 25354^T^ | BAJV00000000 | 38 | 192977 | 3.18 | 55.8 |
| *Azospirillum lipoferum* 59b^T^ | VTTN00000000 | 72 | 372840 | 7.99 | 67.3 |
| *Belnapia moabensis* DSM 16746^T^ | JQKB00000000 | 282 | 89597 | 6.73 | 68.8 |
| *Belnapia rosea* CGMCC 1.10758^T^ | FMXZ00000000 | 106 | 232313 | 6.03 | 69.7 |
| *Bombella apis* SME1^T^ | WHNS00000000 | 11 | 455874 | 2.09 | 59.6 |
| *Bombella favorum* TMW2.1880^T^ | NWUS00000000 | 7 | 1170238 | 1.98 | 55.3 |
| *Bombella intestini* R-52487^T^ | JATM00000000 | 12 | 376854 | 2.02 | 54.9 |
| *Bombella mellum* TMW2.1889^T^ | PDLY00000000 | 11 | 390284 | 2.07 | 60.4 |
| *Caldovatus sediminis* CGMCC 1.16330^T^ | BMKS00000000 | 43 | 364442 | 4.80 | 75.0 |
| *Commensalibacter intestini* A911^T^ | AGFR00000000 | 26 | 476138 | 2.45 | 36.8 |
| *Crenalkalicoccus roseus* YIM 78023^T^ | SJDM00000000 | 127 | 89369 | 4.43 | 73.9 |
| *Dankookia rubra* JCM 30602^T^ | SMSJ00000000 | 458 | 79244 | 7.78 | 70.1 |
| *Elioraea rosea* PF-30^T^ | VFAC00000000 | 95 | 92038 | 4.49 | 69.9 |
| *Elioraea tepidiphila* DSM 17972^T^ | KB899910 | 56 | 144976 | 4.30 | 71.3 |
| *Elioraea thermophila* YIM 72297^T^ | QMDI00000000 | 11 | 685266 | 3.03 | 70.9 |
| *Endobacter medicaginis* CECT 8088^T^ | JACHXV000000000 | 61 | 369956 | 3.63 | 68.2 |
| *Entomobacter blattae* G55GP^T^ | CP060244 | 1 | 2701842 | 2.70 | 45.5 |
| *Gluconacetobacter aggeris* LMG 27801^T^ | JABEQD000000000 | 61 | 257252 | 4.32 | 65.2 |
| *Gluconacetobacter asukensis* LMG 27724^T^ | JABEQE000000000 | 64 | 221504 | 4.39 | 65.2 |
| *Gluconacetobacter azotocaptans* LMG 21311^T^ | JABEQF000000000 | 57 | 382945 | 4.14 | 66.5 |
| *Gluconacetobacter diazotrophicus* LMG 7603^T^ | JABEQG000000000 | 162 | 81054 | 4.17 | 66.2 |
| *Gluconacetobacter dulcium* LMG 1728^T^ | JABEQN00000000 | 76 | 132075 | 4.42 | 64.7 |
| *Gluconacetobacter entanii* LTH 4560^T^ | NKUF00000000 | 157 | 75248 | 3.60 | 62.6 |
| *Gluconacetobacter johannae* LMG 21312^T^ | JABEQH000000000 | 49 | 161760 | 3.63 | 67.3 |
| *Gluconacetobacter liquefaciens* LMG 1382^T^ | JABEQI000000000 | 31 | 336215 | 4.18 | 64.4 |
| *Gluconacetobacter sacchari* LMG 19747 ^T^ | JABEQJ000000000 | 114 | 336232 | 4.83 | 66.0 |
| *Gluconacetobacter takamatsuzukensis* LMG 27800^T^ | JABEQK000000000 | 35 | 133958 | 3.78 | 67.0 |
| *Gluconacetobacter tumulicola* LMG 27725^T^ | JABEQL000000000 | 90 | 258349 | 4.30 | 65.1 |
| *Gluconacetobacter tumulisoli* LMG 27802^T^ | JABEQM000000000 | 49 | 135740 | 3.96 | 66.6 |
| *Gluconobacter albidus* NBRC 3250^T^ | BEWL00000000 | 49 | 240396 | 3.29 | 59.6 |
| *Gluconobacter aidae* AC10^T^ | IPH00000000 | 108 | 84383 | 3.01 | 60.3 |
| *Gluconobacter cerinus* NBRC 3267^T^ | BEWM00000000 | 49 | 575896 | 3.59 | 55.6 |
| *Gluconobacter frateurii* NBRC 3264^T^ | BEWN00000000 | 10 | 734187 | 3.31 | 56.1 |
| *Gluconobacter japonicus* NBRC 3271^T^ | BEWO00000000 | 75 | 140254 | 3.16 | 56.1 |
| *Gluconobacter kanchanaburiensis* NBRC 103587^T^ | BJVA00000000 | 46 | 181223 | 2.93 | 58.4 |
| *Gluconobacter kondonii* NBRC 3266^T^ | BEWP00000000 | 63 | 252441 | 3.27 | 58.3 |
| *Gluconobacter morbifer* G707^T^ | AGQV00000000 | 19 | 422539 | 2.89 | 59.0 |
| *Gluconobacter oxydans* DSM 3503^T^ | SMCY00000000 | 88 | 101229 | 2.92 | 60.8 |
| *Gluconobacter potus* LMG 1764^T^ | LHZB00000000 | 121 | 145531 | 3.64 | 60.5 |
| *Gluconobacter roseus* NBRC 3990^T^ | BJLY00000000 | 12 | 733426 | 2.88 | 59.9 |
| *Gluconobacter sphaericus* NBRC 12467^T^ | BJMK00000000 | 97 | 145601 | 3.06 | 58.2 |
| *Gluconobacter thailandicus* NBRC 100600^T^ | BJWD00000000 | 98 | 108173 | 3.42 | 56.2 |
| *Gluconobacter vitians* LMG 31484^T^ | JABCQG00000000 | 93 | 145741 | 3.17 | 60.0 |
| *Gluconobacter wancherniae* NBRC 103581^T^ | BJUZ00000000 | 9 | 1521677 | 2.78 | 55.3 |
| *Granulibacter bethesdensis* CGDNIH1^T^ | NC_008343 | 1 | 2708434 | 2.71 | 59.1 |
| *Humitalea rosea* DSM 24525^T^ | QKYU00000000 | 73 | 161053 | 4.97 | 69.6 |
| *Komagataeibacter cocois* WE7^T^ | QEXL00000000 | 75 | 168149 | 3.41 | 62.3 |
| *Komagataeibacter diospyri* MSKU9^T^ | BDLU00000000 | 105 | 95112 | 3.76 | 60.4 |
| *Komagataeibacter hansenii* LMG 23726^T^ | NKUD00000000 | 58 | 142792 | 3.48 | 59.6 |
| *Komagataeibacter kakiaceti* DSM 24098^T^ | JACIJA000000000 | 34 | 320974 | 3.39 | 62.9 |
| *Komagataeibacter maltaceti* LMG 1529^T^ | POTC00000000 | 163 | 59924 | 3.63 | 63.2 |
| *Komagataeibacter medellinensis* NBRC 3288^T^ | NC_016027 | 8 | 3136818 | 3.51 | 60.6 |
| *Komagataeibacter melomenusus* AV436^T^ | JABJWC00000000 | 118 | 67849 | 3.64 | 62.9 |
| *Komagataeibacter nataicola* LMG 1536^T^ | NIRT00000000 | 106 | 107486 | 3.67 | 61.5 |
| *Komagataeibacter oboediens* LMG 18849^T^ | NKTX00000000 | 278 | 59803 | 3.78 | 61.4 |
| *Komagataeibacter pomaceti* T5K1^T^ | NOXG00000000 | 138 | 138141 | 3.45 | 62.5 |
| *Komagataeibacter rhaeticus* LMG 22126^T^ | NKTZ00000000 | 64 | 207167 | 3.47 | 63.5 |
| *Komagataeibacter saccharivorans* LMG 1582^T^ | NKTY00000000 | 107 | 106895 | 3.35 | 61.6 |
| *Komagataeibacter sucrofermentans* LMG 18788^T^ | NKUA00000000 | 83 | 145161 | 3.36 | 62.3 |
| *Komagataeibacter swingsii* LMG 22125^T^ | NKUB00000000 | 113 | 134398 | 3.73 | 62.4 |
| *Komagataeibacter xylinus* LMG 1515^T^ | NKUC00000000 | 191 | 72813 | 3.66 | 62.2 |
| *Kozakia baliensis* DSM 14400^T^ | CP014674 | 7 | 2888029 | 3.51 | 57.4 |
| *Lichenicoccus roseus* KEBCLARHB70R^T^ | VCID00000000 | 26 | 700473 | 4.79 | 67.8 |
| *Lichenicola cladoniae* PAMC 26569^T^ | CP053708 | 8 | 4799761 | 6.06 | 64.6 |
| *Neoasaia chiangmaiensis* NBRC 101099^T^ | CP014691 | 1 | 3407860 | 3.41 | 61.5 |
| *Neokomagataea tanensis* AH13^T^ | CP032485 | 2 | 2477279 | 2.58 | 51.9 |
| *Neokomagataea thailandica* NBRC 106555 ^T^ | BCZB00000000 | 83 | 84303 | 2.49 | 52.4 |
| *Oecophyllibacter saccharovorans* Ha5^T^ | CP038143 | 2 | 1938936 | 1.95 | 61.5 |
| *Parasaccharibacter apium* A29^T^ | LMYH00000000 | 27 | 201335 | 2.01 | 59.4 |
| *Rhodovarius crocodyli* CCP-6^T^ | SACL00000000 | 35 | 552246 | 5.28 | 69.3 |
| *Rhodovarius lipocyclicus* CCUG 44693^T^ | JAAABL000000000 | 111 | 83545 | 4.59 | 69.9 |
| *Rhodovastum atsumiense* DSM 21279^T^ | VWPK00000000 | 226 | 104226 | 7.10 | 68.7 |
| *Roseicella frigidaeris* DB1506^T^ | QLIX00000000 | 87 | 187085 | 5.82 | 72.5 |
| *Roseococcus suduntuyensis* DSM 19979^T^ | JACIDJ000000000 | 17 | 757046 | 4.14 | 70.8 |
| *Roseomonas aerilata* DSM 19363^T^ | JONP00000000 | 151 | 202657 | 6.43 | 69.7 |
| *Roseomonas aerophila* NBRC 108923^T^ | JACTVA000000000 | 140 | 88290 | 5.67 | 68.9 |
| *Roseomonas aestuarii* JR169-1-13^T^ | PDOA00000000 | 106 | 245408 | 5.20 | 71.5 |
| *Roseomonas algicola* PeD5^T^ | JAAIKB00000000 | 65 | 452050 | 6.56 | 71.0 |
| *Roseomonas alkaliterrae* DSM 25895^T^ | JACIJE000000000 | 50 | 301642 | 4.24 | 72.7 |
| *Roseomonas bella* CQN31^T^ | QGNA00000000 | 14 | 906722 | 5.94 | 71.5 |
| *Roseomonas cervicalis* ATCC 49957^T^ | GG771274 | 498 | 955436 | 5.10 | 62.7 |
| *Roseomonas frigidaquae* JCM 15073^T^ | JAAVTX000000000 | 20 | 749226 | 6.09 | 70.3 |
| *Roseomonas harenae* CPCC 101081^T^ | WWDL00000000 | 126 | 110178 | 5.30 | 68.7 |
| *Roseomonas mucosa* NCTC 13291^T^ | UGVN00000000 | 4 | 4237410 | 5.00 | 70.3 |
| *Roseomonas nepalensis* S9-3B^T^ | RCZP00000000 | 157 | 123179 | 6.54 | 71.9 |
| *Roseomonas oryzae* KCTC 42542^T^ | VUKA00000000 | 95 | 306338 | 4.68 | 69.0 |
| *Roseomonas oryzicola* KCTC 22478^T^ | JAAVUP000000000 | 52 | 685653 | 5.31 | 71.2 |
| *Roseomonas pecuniae* DSM 25622^T^ | JACIJD000000000 | 92 | 200862 | 4.92 | 71.4 |
| *Roseomonas rhizosphaerae* YW11^T^ | PDNU00000000 | 114 | 124656 | 4.65 | 71.9 |
| *Roseomonas rosea* DSM 14916^T^ | FQZF00000000 | 81 | 151080 | 5.34 | 70.8 |
| *Roseomonas selenitidurans* BU-1^T^ | JAAVNE00000000 | 146 | 96152 | 5.79 | 71.7 |
| *Roseomonas stagni* DSM 19981^T^ | FOSQ00000000 | 44 | 359186 | 6.38 | 70.6 |
| *Roseomonas tokyonensis* K-20^T^ | JAFNJS00000000 | 59 | 632449 | 6.16 | 70.5 |
| *Roseomonas vastitatis* CPCC 101021^T^ | QXGS00000000 | 249 | 164366 | 5.13 | 68.7 |
| *Roseomonas wenyumeiae* Z23^T^ | RFLX00000000 | 196 | 179822 | 6.06 | 68.6 |
| *Rubritepida flocculans* DSM 14296^T^ | AUDH00000000 | 74 | 134898 | 3.83 | 73.4 |
| *Saccharibacter floricola* DSM 15669^T^ | KB899333 | 43 | 169586 | 2.38 | 51.1 |
| *Siccirubricoccus deserti* SYSU D8009^T^ | JACOMF000000000 | 275 | 115287 | 6.34 | 69.8 |
| *Skermanella aerolata* KACC 11604^T^ | AVFK00000000 | 276 | 165927 | 8.53 | 64.1 |
| *Swaminathania salitolerans* NBRC 104436^T^ | BJVC00000000 | 9 | 728662 | 2.93 | 62.9 |
| *Swingsia samuiensis* AH83^T^ | CP038141 | 1 | 2163214 | 2.16 | 45.1 |
| *Tanticharoenia sakaeratensis* NBRC 103193^T^ | BALE00000000 | 94 | 111870 | 3.51 | 64.2 |

**Supplementary Table 2. Classification of *Acetobacteraceae* type species into suprageneric or infrageneric clades.**

| **Clade** | **Species** |
| --- | --- |
| *Eliorea* | *E. rosea* PF-30^T^, *E. tepidiphila* DSM 17972^T^, *E. thermophila* YIM 72297^T^ |
| *Pararosemonas* | *Roseomonas aerilata* DSM 19363^T^, *R. nepalensis* S9-3B^T^, *R. harenae* CPCC101081^T^ *R. rosea* DSM 14916^T^, *R. pecuniae* DSM 25622^T^ |
| *Pseudoroseomonas* | *Roseomonas aerophila* NBRC 198923^T^, *R. aestuarii* JR169-1-13^T^, *R. cervicalis* ATCC 49957^T^, *R. oryzae* KCTC 42542^T^, *R. rhizosphaerae* YW11^T^, *R. vastitatis* CPCC 1011021^T^, *R. wenyumeia Z23^T^* |
| *Rhodovarius* | *Rhodovarius crocodyli* CCP-6^T^, *Rhodovarius lipocyclicus* CCUG 44693^T^, *Roseococcus suduntuyensis* DSM 19979^T^, *Rubritepida flocculans* DSM 14296^T^ |
| *Belnapia* | *Belnapia moabensis* DSM 16746^T^, *Belnapia rosea* CGMCC 1.10758^T^, *Caldovatus sediminis* CGMCC1.16330^T^, *Crenalkalicoccus roseus* YIM 78023^T^, *Dankookia rubra* JCM 30602^T^, *Roseicella frigidaeris* DB1506^T^, *Siccirubricoccus deserti* SYSU D8009^T^ |
| *Neoroseomonas* | *Roseomonas alkaliterrae* DSM 25895^T^, *R. oryzicola* KCTC 22478^T^ |
| *Falsiroseomonas* | *Roseomonas algicola* PeD5^T^, *R. bella* CQN31^T^, *R. frigidaquae* JCM 15073^T^, *R. selenitidurans* BU-1^T^, *R. stagni* DSM 19981^T^, *R. tokyonensis* K-20^T^ |
| *Acidocella* | *Acidiphilium angustum* ATCC 35903^T^, *A. cryptum* F-5^T^, *A. multivorum* AIU301^T^, *Acidocella aminolytica* 101^T^, *A. facilis* ATCC 35904^T^ |
| *Rhodovastum* | *Acidibrevibacterium fodinaquatile* G45-3^T^, *Rhodovastum atsumiense* DSM 21279^T^ |
| *Gluconacetobacter* | *G. aggeris* LMG 27801^T^, *G. asukensis* LMG 27724^T^, *G. azotocaptans* LMG 21311^T^, *G. diazotrophicus* LMG 7603^T^, *G. dulcium* LMG 1728^T^, *G. johannae* LMG 21312^T^, *G. liquefaciens* LMG 1382^T^, *G. sacchari* LMG 19747^T^, *G. takamatsuzukensis* LMG 27800^T^, *G. tumulicola* LMG 27725^T^, *G. tumulisoli* LMG 27802^T^ |
| *Komagataeibacter* clade hansenii | *Gluconacetobacter entanii* LTH 4560^T^, *K. cocois* WE7^T^, *K. hansenii* LMG 23726^T^, *K. maltaceti* LMG 1529^T^, *K. pomaceti* T5K1^T^ |
| *Komagataeibacter* clade xylinus | *K. diospyri* MSKU9^T^, *K. kakiaceti* DSM 24098^T^, *K. medellinensis* NBRC 3288^T^, *K. melomenusus* AV436^T^, *K. nataicola* LMG 1536^T^, *K. oboediens* LMG 18849^T^, *K. rhaeticus* LMG 22126^T^, *K. saccharivorans* LMG 1582^T^, *K. sucrofermentans* LMG 18788^T^, *K. swingsii* LMG 22125^T^, *K. xylinus* LMG 1515^T^ |
| *Acetobacter* clade aceti | *A. aceti* NBRC 14818^T^, *A. conturbans* LMG 1627^T^, *A. estunensis* LMG 1626^T^, *A. fallax* LMG 1636^T^, *A. musti* LMG 30640^T^, *A. nitrogenifigens* DSM 23921^T^, *A. oeni* LMG 21952^T^, *A. sacchari* TBRC11175^T^, *A. sicerae* LMG 1531^T^ |
| *Acetobacter* clade pasteurianus | *A. fabarum* LMG 24244^T^, *A. ghanensis* LMG 23848^T^, *A. lambici* LMG 27439^T^, *A. lovaniensis* LMG 1617^T^, *A. okinawensis* JCM 25146^T^, *A. oryzifermentans* SLV-7^T^, *A. oryzoeni* B6^T^, *A. pasteurianus* LMG 1262^T^, *A. pomorum* LHT 2458^T^, *A. syzygii* NBRC 16604^T^ |
| *Acetobacter* clade orleanensis | *A. cerevisiae* LMG 1625^T^, *A. cibinongensis* NBRC 16605^T^, *A. indonesiensis* NBRC 16471^T^, *A. malorum* LMG 1746^T^, *A. orleanensis* NBRC 13752^T^, *A. orientalis* 21F-2^T^, *A. senegalensis* LMG 23690^T^, *A. thailandicus* LMG 30826^T^, *A. tropicalis* NBRC 16470^T^ |
| *Asaia* | *A. astilbis* JCM 15831^T^, *A. bogorensis* NBRC 16594^T^, *A. platycodi* JCM 25414^T^, *A. prunellae* JCM 25354^T^ |
| *Bombella* | *B. apis* SME1^T^, *B. favorum* TMW2.1880^T^, *B. intestini* R-52487^T^, *B. mellum* TMW2.1889^T^, *Parasaccharibacter apium* A29^T^ |
| *Gluconobacter* clade oxydans | *G. albidus* NBRC 3250^T^, *G. aidae* AC10^T^, *G. kanchanaburiensis* NBRC 103587^T^, *G. kondonii* NBRC 3266^T^, *G. potus* LMG 1764^T^,  *G. oxydans* DSM 3503^T^, *G. roseus* NBRC 3990^T^, *G. sphaericus* NBRC 12467^T^, *G. vitians* LMG 31484^T^ |
| *Gluconobacter* clade cerinus | *G. cerinus* NBRC 3267^T^, *G. frateurii* NBRC 3264^T^, *G. japonicus* NBRC 3271^T^, *G. thailandicus* NBRC 100600^T^ |

**
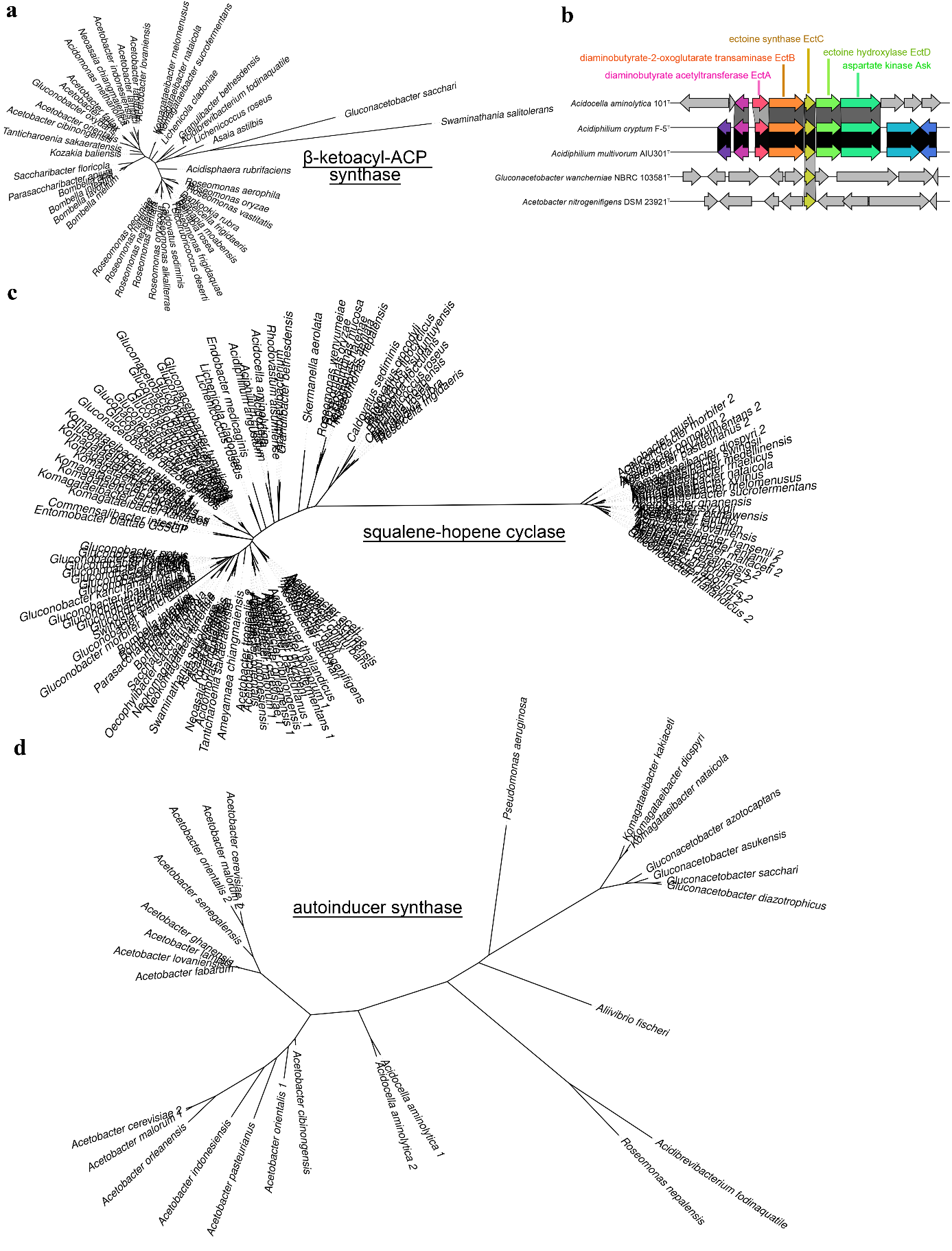
**

**Supplementary Figure 1. Aryl polyene, ectoine, hopanoid and lactone biosynthetic gene cluster in the family *Acetobacteraceae*. a** Unrooted tree based on β-ketoacyl ACP synthase, the central enzyme involved in the biosynthesis of aryl polyenes. **b** Organization of the ectoine biosynthetic gene cluster in the five type strains of the family presumably able to produce ectoines. **c** Unrooted tree based on squalene-hopene cyclase, the central enzyme involved in the biosynthesis of hopane triterpenoids. **d** Unrooted tree based on the autoinducer synthase (LuxI homolog) involved in the biosynthesis of acyl-homoserine lactones.

**
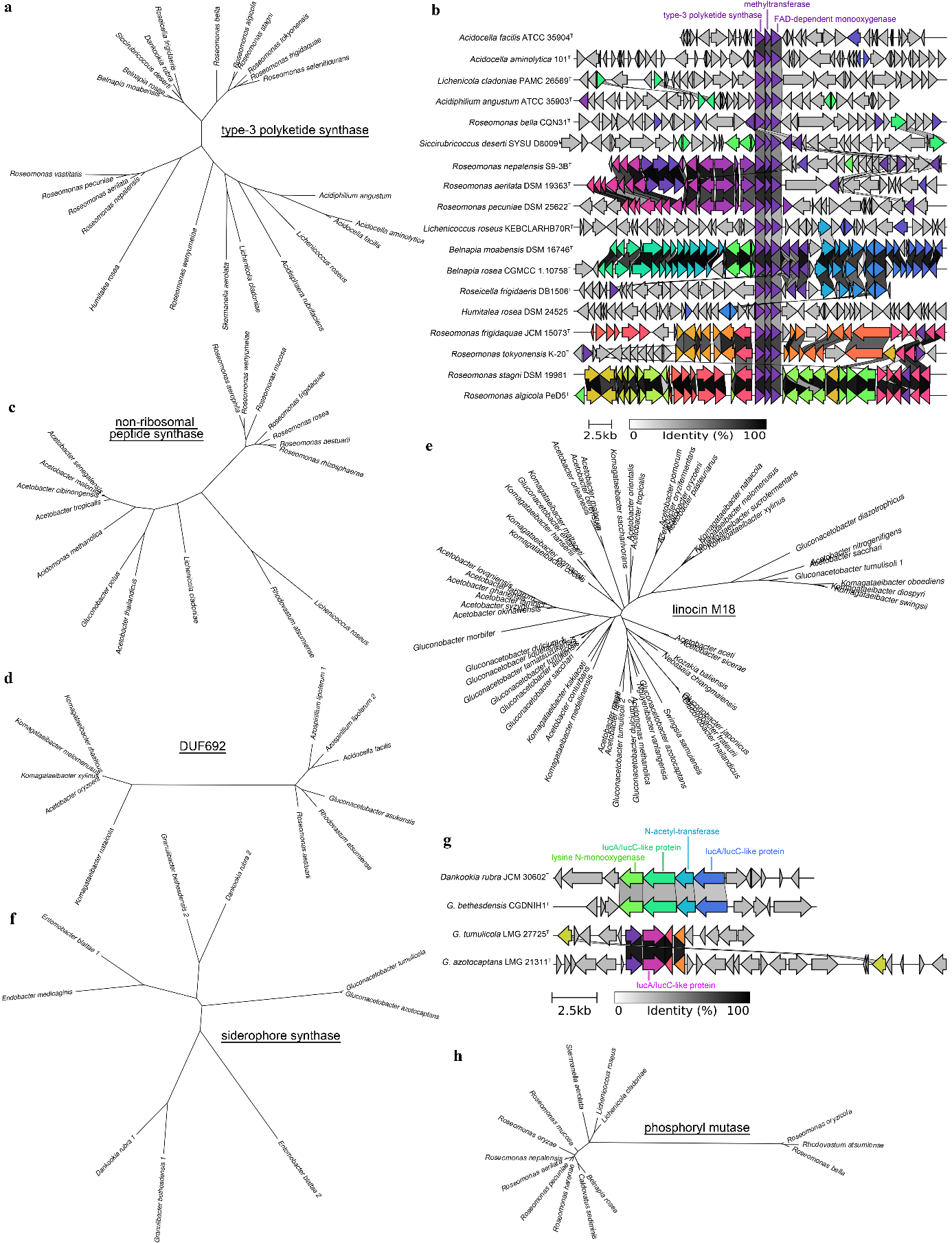
**

**Supplementary Figure 2. Type-3 polyketide, non-ribosomal peptide synthases, ribosomally synthetized and post-translationally modified peptide, siderophore and phosphonate biosynthetic gene cluster in *Acetobacteraceae*. a** Unrooted tree based on type-3 PKS. **b** Organization of type-3 PKS biosynthetic gene clusters. **c** Unrooted tree based on NRPSs containing complete C-A-PCP-TE domains. **d** Unrooted tree based on DUF692 protein. **e** Unrooted tree based on linocin M18 protein. **f** Unrooted tree based on the siderophore synthetase (IucA/IucC-like). **g** Organization of certain siderophore biosynthetic gene clusters. **h** Unrooted tree based on phosphoryl mutase.
